# Supplementary material for: Direct extraction of signal and noise correlations from two-photon calcium imaging of ensemble neuronal activity
Source: eLife. 2021 Jun 28;10:e68046. doi: 10.7554/eLife.68046 (PMC8354639; doi:10.7554/eLife.68046)
Supplement: Transparent reporting form [file elife-68046-transrepform.docx]

***eLife’s* transparent reporting form**

We encourage authors to provide detailed information *within their submission* to facilitate the interpretation and replication of experiments. Authors can upload supporting documentation to indicate the use of appropriate reporting guidelines for health-related research (see [EQUATOR Network](http://www.equator-network.org/%20)), life science research (see the [BioSharing Information Resource](https://biosharing.org/)), or the [ARRIVE guidelines](http://www.plosbiology.org/article/info:doi/10.1371/journal.pbio.1000412) for reporting work involving animal research. Where applicable, authors should refer to any relevant reporting standards documents in this form.

If you have any questions, please consult our Journal Policies and/or contact us: [editorial@elifesciences.org](mailto:editorial@elifesciences.org).

**Sample-size estimation**

- You should state whether an appropriate sample size was computed when the study was being designed
- You should state the statistical method of sample size computation and any required assumptions
- If no explicit power analysis was used, you should describe how you decided what sample (replicate) size (number) to use

Please outline where this information can be found within the submission (e.g., sections or figure legends), or explain why this information doesn’t apply to your submission:

The data used in this work served to validate the tools developed in terms of estimation accuracy. As such, the hypotheses tested did not require explicit power analysis to determine sample-size. However, our theoretical analysis (Theorem 1, pages 16) provides a quantification of the bias and variance of the estimators, which can be used as a guideline for sample-size estimation in follow-up studies.

**Replicates**

- You should report how often each experiment was performed
- You should include a definition of biological versus technical replication
- The data obtained should be provided and sufficient information should be provided to indicate the number of independent biological and/or technical replicates
- If you encountered any outliers, you should describe how these were handled
- Criteria for exclusion/inclusion of data should be clearly stated
- High-throughput sequence data should be uploaded before submission, with a private link for reviewers provided (these are available from both GEO and ArrayExpress)

Please outline where this information can be found within the submission (e.g., sections or figure legends), or explain why this information doesn’t apply to your submission:

The number of trials, neurons and time samples considered in each simulation study and real data study are indicated in the corresponding Results sections as well as in Methods and Materials under subsections Guidelines for model parameter settings (pages 24) and Experimental procedures (pages 29).

**Statistical reporting**

- Statistical analysis methods should be described and justified
- Raw data should be presented in figures whenever informative to do so (typically when N per group is less than 10)
- For each experiment, you should identify the statistical tests used, exact values of N, definitions of center, methods of multiple test correction, and dispersion and precision measures (e.g., mean, median, SD, SEM, confidence intervals; and, for the major substantive results, a measure of effect size (e.g., Pearson's r, Cohen's d)
- Report exact p-values wherever possible alongside the summary statistics and 95% confidence intervals. These should be reported for all key questions and not only when the p-value is less than 0.05.

Please outline where this information can be found within the submission (e.g., sections or figure legends), or explain why this information doesn’t apply to your submission:

Details of the specific statistical tests used are given in Methods and Materials under subsection Performance evaluation (pages 27). Test statistics are reported in Table 1 (page 9), Table 2 (page 13), Table 3 (page 15), as well as in the insets of Fig. 4 (page 9), Fig. 5 (page 11), Fig. 7 (page 14), Fig. 2–Suppl. 5 & 6 (pages 54-55), Fig. 4–Suppl. 1 (page 56), Fig. 6–Suppl. 1 (page 57), Fig. 7–Suppl. 1 & 2 (pages 57-58).

(For large datasets, or papers with a very large number of statistical tests, you may upload a single table file with tests, Ns, etc., with reference to sections in the manuscript.)

**Group allocation**

- Indicate how samples were allocated into experimental groups (in the case of clinical studies, please specify allocation to treatment method); if randomization was used, please also state if restricted randomization was applied
- Indicate if masking was used during group allocation, data collection and/or data analysis

Please outline where this information can be found within the submission (e.g., sections or figure legends), or explain why this information doesn’t apply to your submission:

Group allocation is not applicable to this study, as the hypotheses tested did not require using separate control and treatment groups. Methods of extracting fluorescence activity of individual cells from two-photon images are described in Methods and Materials under subsection Experimental procedures (page 29).

**Additional data files (“source data”)**

- We encourage you to upload relevant additional data files, such as numerical data that are represented as a graph in a figure, or as a summary table
- Where provided, these should be in the most useful format, and they can be uploaded as “Source data” files linked to a main figure or table
- Include model definition files including the full list of parameters used
- Include code used for data analysis (e.g., R, MatLab)
- Avoid stating that data files are “available upon request”

Please indicate the figures or tables for which source data files have been provided:

The data used in this work is publicly available in the Digital Repository at the University of Maryland at <http://hdl.handle.net/1903/26917>. The codes have been made publicly available in GitHub at <https://github.com/Anuththara-Rupasinghe/Signal-Noise-Correlation>. Both repositories are cited in the manuscript.
